# The Space Between: Exploring Perivascular Pathways of Alzheimer’s Disease Risk Detection

**DOI:** 10.1002/alz70861_108149

**Published:** 2025-12-23

**Authors:** Lauren Abigail Houchin, Jenna N. Adams, Soyun Kim, Lisa M. Taylor, Mithra Sathishkumar, Liv McMillan, Alyssa L. Lawrence, Niels Janssen, Michael A Yassa

**Affiliations:** ^1^ University of California, Irvine, Irvine, CA USA; ^2^ Universidad de La Laguna, Santa Cruz de Tenerife Spain

## Abstract

**Background:**

Perivascular spaces (PVS) are small, fluid‐filled spaces surrounding cerebral blood vessels which facilitate metabolic transport and waste clearance. When this clearance process is disrupted, PVS become visible on magnetic resonance imaging, signaling cerebrovascular injury. Recent studies suggest PVS may be associated with cognitive decline and Alzheimer’s disease (AD) diagnosis. Our study examines the relationship between MRI‐visible PVS, memory performance, neurodegeneration, and AD biomarkers among a preclinical population of older adults.

**Method:**

We included 120 participants from the Biomarker Exploration in Aging, Cognition, and Neurodegeneration (BEACoN) study (66.6% female, mean age = 72.19). PVS segmentation was performed on T1‐weighted MRI using a deep learning algorithm, generating whole‐brain and regional (basal ganglia [BG], medial temporal lobe [MTL], and centrum semiovale [CSO]) PVS volumes and counts, normalized for volume. We assessed associations between PVS and (1) memory performance (Rey Auditory Verbal Learning Test [RAVLT] delayed recall), (2) neurodegeneration (hippocampal volume derived from FreeSurfer segmentation), and (3) AD pathology (18F‐florbetapir‐PET amyloid‐beta positivity [Aβ] and plasma *p* ‐tau217) using partial correlations, covarying for age, sex, and education.

**Result:**

Whole‐brain PVS volume and count were not associated with RAVLT delayed recall, Aβ positivity, or *p* ‐tau217 levels. However, whole‐brain PVS count was significantly negatively correlated with hippocampal volume (r= –.198, *p* = 0.039). Regionally, PVS were detected in the putamen, pallidum, and caudate, but not in the MTL or nucleus accumbens, suggesting regionally specific PVS expansion. Total PVS volume within the BG showed a negative trending relationship with RAVLT delayed recall (*p* = 0.08), while total PVS count in the BG was positively correlated with *p* ‐tau217 levels (r= .448, *p* = 0.019). BG PVS count was additionally negatively correlated with hippocampal volume (r= –0.303, *p* = 0.01), but this effect was reduced after controlling for covariates (*p* = 0.09). Notably, despite similar PVS burden, the CSO did not show associations with memory performance, hippocampal volume, or *p* ‐tau217.

**Conclusion:**

Our findings suggest that PVS may serve as a marker of early neurodegenerative changes in memory performance, hippocampal structure, and AD pathology, and may be a sensitive biomarker of AD risk in preclinical populations. Future work will examine spatial relationships between PVS and other white matter pathologies.

